# Acetic Acid Ketonization over Fe_3_O_4_/SiO_2_ for Pyrolysis Bio‐Oil Upgrading

**DOI:** 10.1002/cctc.201601269

**Published:** 2017-01-18

**Authors:** James A. Bennett, Christopher M. A. Parlett, Mark A. Isaacs, Lee J. Durndell, Luca Olivi, Adam F. Lee, Karen Wilson

**Affiliations:** ^1^ European Bioenergy Research InstituteAston UniversityBirminghamB4 7ETUK; ^2^ Elettra Sincrotrone Trieste34149BasovizzaItaly

**Keywords:** carboxylic acids, iron, nanoparticles, supported catalysts, waste prevention

## Abstract

A family of silica‐supported, magnetite nanoparticle catalysts was synthesised and investigated for continuous‐flow acetic acid ketonisation as a model pyrolysis bio‐oil upgrading reaction. The physico‐chemical properties of Fe_3_O_4_/SiO_2_ catalysts were characterised by using high‐resolution transmission electron microscopy, X‐ray absorption spectroscopy, X‐ray photo‐electron spectroscopy, diffuse reflectance infrared Fourier transform spectroscopy, thermogravimetric analysis and porosimetry. The acid site densities were inversely proportional to the Fe_3_O_4_ particle size, although the acid strength and Lewis character were size‐invariant, and correlated with the specific activity for the vapour‐phase acetic ketonisation to acetone. A constant activation energy (∼110 kJ mol^−1^), turnover frequency (∼13 h^−1^) and selectivity to acetone of 60 % were observed for ketonisation across the catalyst series, which implies that Fe_3_O_4_ is the principal active component of Red Mud waste.

## Introduction

**Scheme 1:**
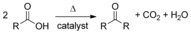
Carboxylic acid ketonization.

Bio‐oil is a renewable (and potentially sustainable) liquid fuel prepared by the pyrolysis of biomass feedstocks such as agricultural or forestry waste, energy crops or microalgae solid residues and sewage sludge.^1^ The direct use of unprocessed fast pyrolysis bio‐oils is hindered by their undesirable physico‐chemical properties, which include a low heating value because of their high oxygen content, high viscosity and high acidity, which renders it corrosive and (thermo‐)chemically unstable.^2^ The latter arises from the presence of significant concentrations of carboxylic acids formed during the thermal decomposition of cellulose and hemicellulose biomass components to lead to acetic acid at levels between 1–10 %. Heterogeneous catalysis affords several routes for the upgrading of pyrolysis bio‐oils, which include esterification,^3^ aldol condensation,^4^ hydrodeoxygenation (HDO)^5^ and ketonisation,^6^ each of which offer advantages and drawbacks. The esterification of bio‐oil condensates over solid Brønsted acids can afford the low‐temperature liquid‐phase upgrading of the aqueous bio‐oil fraction^7^ but requires a sustainable alcohol source (although self‐esterification with phenolic bio‐oil components is possible) and only slightly lowers the oxygen content. Aldol condensation over solid bases enables chain growth and improves oil stability by removing reactive oxygenate components but does not neutralise the intrinsic acidity that indeed induces catalyst deactivation. HDO is an effective means to obtain cyclic and aliphatic alkanes as drop‐in transportation bio‐fuels, however, this requires a sustainable source of H_2_, and the metal component of HDO catalysts is susceptible to leaching in acidic bio‐oils, hence their neutralisation should help to minimise precious metal usage. Ketonisation, through the condensation of two carboxylic acid molecules to form a heavier ketone with the elimination of CO_2_ and water (Scheme 1), affords a facile means to reduce the acidity and oxygen content of pyrolysis vapour (through close‐coupling to a pyrolysis unit) or the associated bio‐oil condensate simultaneously. For a monocarboxylic acid (RCOOH), such as acetic acid, ketonisation decreases the oxygen content by 75 % and increases the chain length by (R−1) carbon atoms.

Metal oxides, which include iron oxides,^8^ a major component of Red Mud, have been demonstrated widely as active catalysts for ketonisation.^9^ Red Mud is an industrial waste material from bauxite mining for aluminium production^10^ that comprises a toxic and caustic mixture of transition, alkali and alkali earth metal oxides. Generally, such waste is sent to landfill, and hence in conjunction with the scale (120 million tons per annum) of the production of this hazardous material, additional opportunities are sought to add value to Red Mud waste streams.^11^ Consequently, there are several reports of potential processes that address the valorisation of Red Mud, which include its use in construction,^12^ wastewater treatment,^13^ the preparation of geo‐polymers^14^ and magnetic materials,^15^ energy storage^16^ and catalysis for diverse transformations, such as bio‐diesel production,^17^ biomass pyrolysis,^18^ oxidation,^19^ hydrogen production^20^ and the upgrading of fast pyrolysis bio‐oils.^21^ Hematite, α‐Fe_2_O_3_, is a major catalytically active component of Red Mud, which constitutes typically 30–50 wt %,^22^ and has been investigated for the ketonisation of formic and acetic acid mixtures as model reactions for the upgrading of pyrolysis bio‐oils. The hematite present in Red Mud is reported to reduce to ferromagnetic Fe_3_O_4_ during reactions >350 °C.^21^ This reduced mixture is itself catalytically active and exhibits a better selectivity than the parent Red Mud with a 10–20 % higher ketone selectivity.^21^, ^22^ Acetic acid ketonisation over bulk hematite is also reported to induce in situ catalyst reduction to Fe_3_O_4_, which is proposed to exhibit a superior activity to Fe_2_O_3_.^23^ Indeed, Taimoor et al. reported that Fe_2_O_3_ ketonisation activity was enhanced upon the addition of 50 vol % H_2_ to the feedstream,^8^ although direct evidence for Fe_3_O_4_ formation was not provided. Nevertheless, the consensus is that magnetite is probably the stable, and catalytically active, iron oxide phase present during ketonisation.

The mechanism(s) of heterogeneously catalysed carboxylic acid ketonisation and the associated rate‐determining step(s) have yet to be established unequivocally,^6^, ^24^ and a range of reactive intermediates, such as ketenes, enols, acyl carbonium ions, acid anhydrides and β‐keto acids have been invoked. However, there is agreement that adsorbed carboxylate ions are required, and an α‐hydrogen atom must be present on at least one of the reacting acid functions.24a, 25 The barrier to the abstraction of this α‐hydrogen atom by lattice oxygen over a monoclinic ZrO_2_(1 1 1) surface was calculated by using DFT to be 120–159 kJ mol^−1^, which depends on the degree of branching at the α‐carbon atom,^26^ similar to the activation energy for acetic acid ketonisation over ZrO_2_ derived experimentally of 117 kJ mol^−1^.25b This correlation suggests that α‐hydrogen abstraction may be rate‐determining, as proposed for acid ketonisation over CeO_2_ 27 and TiO_2_.^28^ However, condensation and decarboxylation steps have also been proposed to be limiting,25b and there is evidence for a bimolecular rate‐determining step in which adsorbed carboxylate is attacked by enolate to form a β‐keto acid intermediate.^29^ Generally, these mechanisms invoke the dissociative adsorption of a carboxylic acid as a carboxylate over a Lewis acid site, and the carboxylate conjugate proton is bound at a neighbouring lattice oxygen Lewis base site. A second Lewis acid centre adjacent to the first is proposed to activate the second carboxylic acid molecule and their subsequent coupling. Carboxylic acid ketonisation has been reviewed extensively elsewhere.^6^

The dimensions of Fe_3_O_4_ nanoparticles are well known to affect their magnetic,^30^ electrical^31^ and rheological^32^ properties and photo‐activity.^33^ However, size effects have never been investigated in iron oxide catalysed ketonisation. Here we explore structure–reactivity relationships for the vapour‐phase ketonisation of acetic acid over silica‐supported magnetite nanoparticles under continuous‐flow conditions.

## Results and Discussion

**Figure 1:**
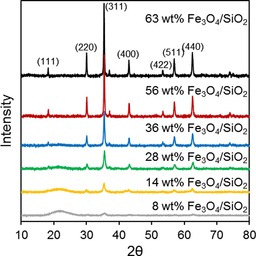
XRD patterns of Fe_3_O_4_/SiO_2_ as a function of Fe loading.

A family of Fe_3_O_4_ catalysts of varying particle size was prepared by dispersing iron oxide over fumed silica at different loadings and characterised by using bulk and surface analytical techniques. The XRD patterns exhibited reflections characteristic of magnetite crystallites in all cases (Figure 1; JCPDS #75‐0033), the peak intensities and widths of which increased and decreased with the Fe_3_O_4_ loading (the weak, broad reflection centred around 2 *θ*=21° arises from the fumed silica support). Peak width analysis using the Scherrer equation revealed a continuous increase in the volume‐averaged Fe_3_O_4_ crystallite diameters from 6 to 45 nm across the family (Table 1), consistent with the corresponding mean particle sizes determined by using TEM (Table 1 and Figure S1); TEM also showed a similar, quasi‐spherical morphology for the magnetite particles independent of the iron oxide loading (Figure S1). N_2_ porosimetry evidenced type II isotherms indicative of microporous fumed silicas^34^ for all materials (Figure S2), and the BET surface areas decreased monotonically with Fe_3_O_4_ loading (Table 1) presumably associated with micropore blockage. The acid site densities of the materials were proportional to their estimated Fe_3_O_4_ surface areas (Table S1) calculated by assuming spherical particles with diameters obtained by using XRD (Table 1). This reveals a maximum for 28 wt % Fe_3_O_4_, which reflects the balance between the competing influences of Fe_3_O_4_ loading and particle size on the associated surface area and hence acid density.

**Table 1:** Physico‐chemical properties of Fe_3_O_4_/SiO_2_ catalysts.

Catalyst^[a]^	Particle size	Surface area^[d]^	Acid
	[nm]	[m^2^ g^−1^]	density^[e]^
Fumed SiO_2_	–	280	–
4.0 wt % Fe_3_O_4_/SiO_2_	6.1^[b]^ (6.0)^[c]^	225	0.169
8.1 wt % Fe_3_O_4_/SiO_2_	9.7 (11.0)	234	0.199
14.4 wt % Fe_3_O_4_/SiO_2_	16.6 (16.6)	218	0.256
28.0 wt % Fe_3_O_4_/SiO_2_	18.1 (17.0)	207	0.288
36.3 wt % Fe_3_O_4_/SiO_2_	27.8 (27.0)	153	0.220
55.9 wt % Fe_3_O_4_/SiO_2_	38.9 (40.0)	124	0.251
63.4 wt % Fe_3_O_4_/SiO_2_	44.7 (46.0)	103	0.252

[a] Fe loadings obtained by using ICP‐OES. [b] XRD. [c] HRTEM. [d] BET. [e] Propylamine TGA–MS.

As magnetite and maghemite (γ‐Fe_2_O_3_) are both inverse spinel structures with similar diffraction patterns and d‐spacings, confirmation of the supported iron oxide phase was sought by using X‐ray absorption spectroscopy (XAS). The common iron oxide phases (α‐Fe_2_O_3_, γ‐Fe_2_O_3_, Fe_3_O_4_ and FeO) all exhibit similar, but readily distinguishable K‐edge X‐ray absorption near edge structure (XANES)^35^ with characteristic pre‐edge and shoulder features caused by 1s→4s and 1s→3d transitions, respectively.

**Figure 2:**
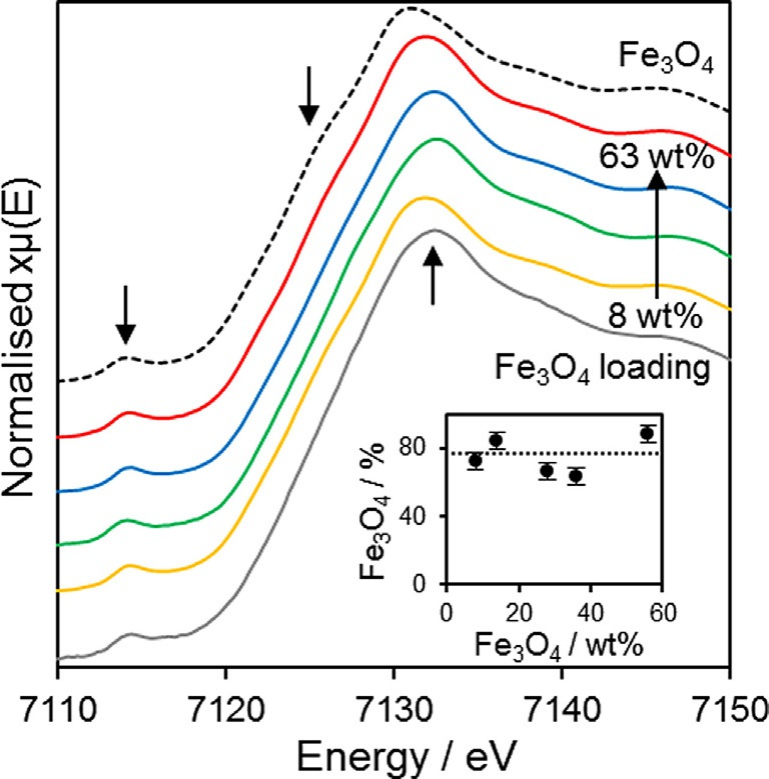
Normalised Fe K‐edge transmission XAS of Fe_3_O_4_/SiO_2_ as a function of Fe loading.

The shape, position and intensity of these features and the absorption edge (white line) are influenced by site geometry, oxidation state and bond length, for which higher oxidation states shift absorption features to higher energy; for Fe^3+^ and Fe^2+^ in similar environments this shift is ∼2–3 eV,35a, 36 and the K‐edge white line increases in the order FeO<Fe_3_O_4_<Fe_2_O_3_ because of a higher 1s electron binding energy (BE) and the shortening of the Fe−O bond. Normalised XANES spectra of all Fe_3_O_4_/SiO_2_ materials resembled that of a pure Fe_3_O_4_ standard closely (Figure 2) and exhibited common pre‐edge, shoulder and white line features at BE=7113, 7124 and 7129 eV, respectively, almost identical to those of pure Fe_3_O_4_. Linear combination fitting of the Fe_3_O_4_/SiO_2_ spectra to FeO, Fe_3_O_4_, Fe_2_O_3_ and metallic Fe standards confirmed that at least 75 % of the iron oxide in all the supported materials was present as Fe_3_O_4_ (Figure 2, inset).

The nature of the supported iron oxide phase and its surface concentration was studied by using X‐ray photo‐electron spectroscopy (XPS; Figure S3). Multiplet splitting, caused by crystal field splitting and shake‐up processes, influences the 2p XPS spectra of many 3d transition metals strongly;^37^ Fe^3+^ and high‐spin Fe^2+^ possess unpaired d electrons and hence their 2p XPS spectra exhibit multiplet splitting.^37^, ^38^ The 2p XPS spectra of the present Fe_3_O_4_/SiO_2_ family all exhibited broad 2p_3/2_ and 2p_1/2_ spin–orbit split multiplets centred around BE=710 and 723 eV, respectively. Theoretical37a and experimental38a studies of Fe_3_O_4_ demonstrate that the 2p_3/2_ region requires fitting with seven components; two of which arise from high‐spin Fe^2+^, and the other five peaks are from Fe^3+^. Our XPS spectra exhibited an excellent fit to the multiplet components of Fe_3_O_4_ (an example for 63 wt % Fe_3_O_4_/SiO_2_ is shown in Figure S4) with a fitted Fe^3+^/Fe^2+^ intensity ratio of 2.08:1 almost identical to that predicted for stoichiometric Fe_3_O_4_. The same stoichiometry was obtained by fitting the Fe 2p XPS spectra of all Fe_3_O_4_/SiO_2_ catalysts. All three X‐ray methods thus confirmed the synthesis of a family of (almost) pure Fe_3_O_4_ nanoparticles dispersed over silica with sizes that increased systematically.

**Figure 3:**
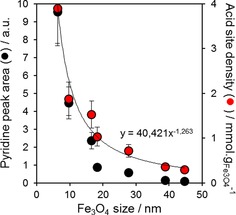
Surface acidity of Fe_3_O_4_/SiO_2_ as a function of particle size. Lewis acid ($\bar{\nu }$ =1445 cm^−1^) band intensities after pyridine titration and acid densities derived from reactively formed propene desorption after propylamine titration are shown normalised to the mass of Fe_3_O_4_ in each sample.

Ketonisation is believed widely to proceed through the adsorption of carboxylate anions at acid sites,6b hence the acid properties of Fe_3_O_4_/SiO_2_ materials were probed by using pyridine titration. The resulting diffuse reflectance infrared Fourier transform spectroscopy (DRIFTS) spectra (Figure S5) only exhibited vibrational bands attributable to pyridine coordinated to Lewis acid sites at $\bar{\nu }$ =1447 and 1599 cm^−1[39]^ for all Fe_3_O_4_ particle sizes, and the band intensities were inversely proportional to size (loading), which indicates that small particles possess a higher acidity. The surface acid density of supported Fe_3_O_4_ nanoparticles was confirmed by using independent qualitative pyridine (DRIFTS) and quantitative propylamine (temperature‐programmed reaction spectroscopy (TPRS); Figure S6) titrations, normalised per mass of Fe_3_O_4_, and was inversely proportional to the particle diameter with a proportionality constant close to unity (Figure 3). This suggests that the acidity of our Fe_3_O_4_/SiO_2_ materials is dictated predominantly by the geometric surface area of the iron oxide, which reflects their common pure Lewis character, and structural and electronic properties observed by using XRD, XAS and XPS. The acid densities of Fe_3_O_4_/SiO_2_ presented in Figure 3 compare very favourably with that of bulk magnetite (0.01–0.02 mmol g^−1^)^40^ and are similar to those of Fe_2_O_3_ supported on mesoporous silica^41^ and mesoporous ZSM‐5^42^ of 1.28–10.4 and 1.3–11 mmol g${}_{\mathrm{FeO}{}_{x}}$ ^−1^, respectively. Some evidence for a slight increase in acid strength with particle size is apparent from a small decrease in the desorption temperature for reactively formed propene at ∼400 °C evolved following propylamine adsorption (Figure S6), which is characteristic of weak/moderate‐strength acid sites.

**Figure 4:**
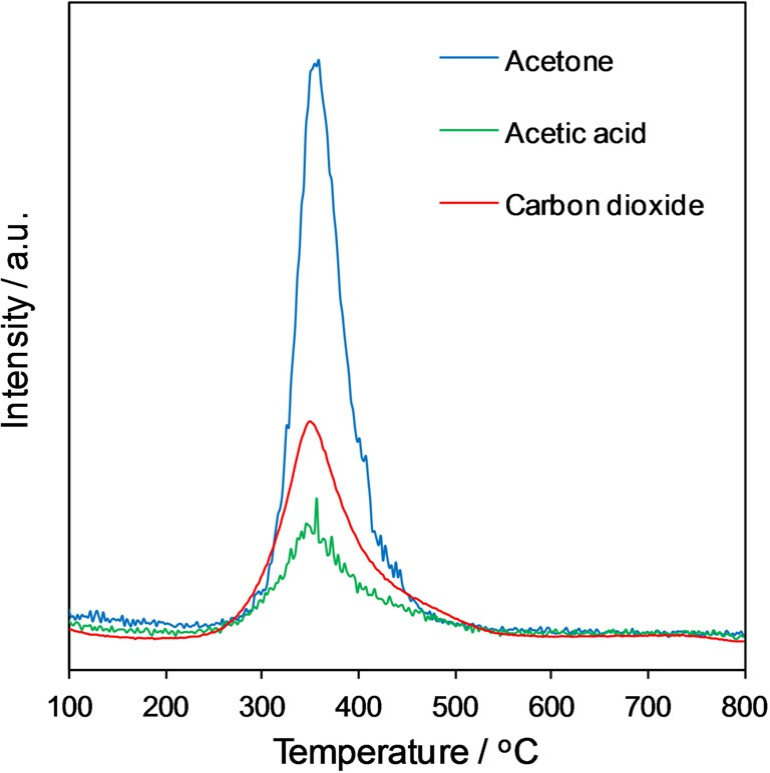
TPRS spectra from acetic acid saturated 4 wt % Fe_3_O_4_/SiO_2_ that show the coincident evolution of the ketonisation products acetone (*m*/*z* 58) and CO_2_ (*m*/*z* 44).

Acetic acid adsorption over Fe_3_O_4_/SiO_2_ was explored subsequently by using DRIFTS to investigate the nature and strength of resultant adsorbed acetate (Figure S7). Samples were pre‐saturated with acetic acid and heated to remove physisorbed species. Spectra with bands at $\bar{\nu }$ =1535, 1445 and 1350 cm^−1^, characteristic of *ν*_as_(COO^−^), *ν*_sym_(COO^−^) and *δ*_s_(CH_3_) modes of bidentate acetate groups adsorbed over metal oxides, respectively, were observed for all Fe_3_O_4_ particle sizes. The frequency difference of 90 cm^−1^ between the *ν*_as_(COO^−^) and *ν*_sym_(COO^−^) stretches indicates a bidentate chelating carboxylate geometry,^43^ which contrasts to that reported for acetic acid over ZrO_2_ and TiO_2_,^44^ for which a bidentate bridging geometry appears to be favoured. Acetate vibrational band intensities were proportional to the Fe_3_O_4_ surface area. We performed thermogravimetric analysis (TGA) with MS of the same acetic acid saturated Fe_3_O_4_/SiO_2_ samples to reveal coincident, reaction‐rate‐limited desorption of reactively formed acetone and CO_2_ (Figure 4) alongside competitive acetic acid desorption. The desorption temperatures and associated apparent activation energies for the evolution of reactively formed acetone and CO_2_ were independent of the Fe_3_O_4_ particle size, and the apparent activation energy was approximately 120 kJ mol^−1^ consistent with that obtained from continuous‐flow ketonisation as described below, which implies a common active site. Notably, a 1:1 molar stoichiometry of acetone/CO_2_ products is expected, close to the ratio observed by using TGA–MS (Figure 4) if the higher electron‐impact ionisation cross‐section of acetone (∼2.5 times that of CO_2_ at 100 eV) is taken into account.

**Figure 5:**
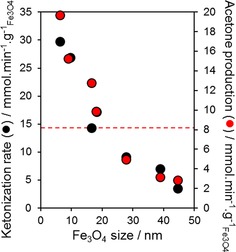
Mass‐normalised rates of acetic acid ketonisation and acetone production as a function of particle size at 400 °C. Dashed lines indicate the corresponding rates of Red Mud.

The catalytic performance of Fe_3_O_4_/SiO_2_ materials was evaluated in the continuous‐flow ketonisation of acetic acid, the major acid component of fast pyrolysis oil, which requires upgrading to reduce the oxygen content and improve bio‐oil stability.^3^, ^45^ Typical reported reaction temperatures of between 300–450 °C afforded steady‐state acetic acid conversions of 30–95 % (Figure S8), and both conversion and steady‐state activity (Figure S9) increase with temperature but are inversely proportional to the Fe_3_O_4_ particle size. The apparent activation energies (calculated for acetic acid conversion <50 % in all cases) were size‐invariant and 100–116 kJ mol^−1^ (Figure S10) consistent with reported values for continuous acetic acid ketonisation over iron oxides (101^8^ and 65–140 kJ mol^−1[23b]^ over γ‐Fe_2_O_3_) and related metal oxides (117 kJ mol^−1^ for ZrO_2_,25b 78–161 kJ mol^−1^ over CeO_2_ 46 and 160 kJ mol^−1^ for Ru/TiO_2_).^47^ The corresponding rates of acetic acid conversion and acetone production, normalised to the Fe_3_O_4_ mass, at 400 °C are compared in Figure 5 and confirm the superior reactivity of small Fe_3_O_4_ nanoparticles, in agreement with their higher mass‐normalised acid site density (Figure 3). Interestingly, the rates of acetic acid conversion/acetone production over an industrial Red Mud waste sample lie approximately in the middle of values for Fe_3_O_4_/SiO_2_, which indicates that Fe_3_O_4_ is likely the principal active component of Red Mud. (Notably, our Red Mud sample contained 25–40 nm Fe_3_O_4_ nanoparticles after ketonisation at 450 °C).

The direct comparison of our Fe_3_O_4_/SiO_2_ ketonisation activity with reported metal oxide catalysts is hindered by the wide range of reactor designs and operating conditions employed and a general focus on acetic acid conversion rather than activity. However, the present acetic acid ketonisation rates of 1.2–4.8 mmol min^−1^ g_catalyst_^−1^ (the maximum value is 18.3 wt % for Fe_3_O_4_/SiO_2_) compare favourably with values of ≈0.5 mmol min^−1^ g_catalyst_^−1^ reported for 5 wt % Ru/TiO_2_ 47 under flow conditions and 0.2–2.4 mmol min^−1^ g_catalyst_^−1^ for CeO_2_ catalysts under batch conditions,^46^ although a rate of 100 mmol min^−1^ g_catalyst_^−1^ has been claimed over strong base sites in polycrystalline pure magnesia under flow conditions.^48^ The XRD patterns of spent reference α‐Fe_2_O_3_, γ‐Fe_2_O_3_ and FeO phases after acetic acid ketonisation under our reaction conditions reveal their respective in situ reduction or oxidation to Fe_3_O_4_ (Figure S12), which indicates these other iron oxide phases are simply precursors to a common magnetite active phase. Acetone does not adsorb strongly on magnetite, as evidenced from the use of temperature‐programmed DRIFTS studies of the acetone‐saturated oxide (Figure S13), in which no characteristic acetone bands were visible >50 °C, and hence acetone is expected to desorb rapidly upon formation at 400 °C. Although acetone oxidation may be possible at high temperatures over iron oxides,^49^ such chemistry is not expected in the present study in which ketonisation was performed by employing N_2_ as the carrier gas.

**Figure 6:**
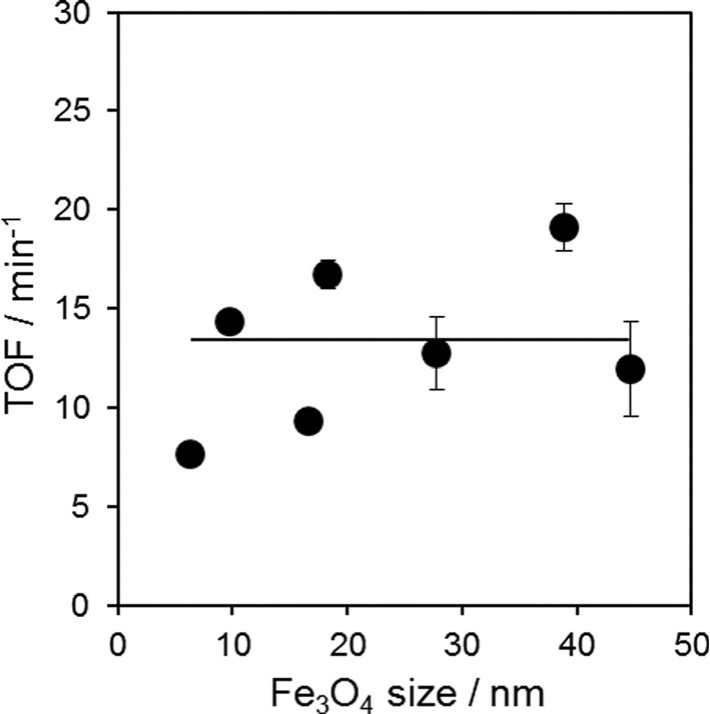
TOFs per acid site for acetic acid ketonisation over Fe_3_O_4_/SiO_2_ as a function of the particle size at 400 °C.

The turnover frequencies (TOFs) per surface acid site are shown in Figure 6 and reveal that acetic acid ketonisation is structure‐insensitive over Fe_3_O_4_ for nanoparticles of 6–60 nm, which is anticipated in light of their size‐invariant acid strength/character and common activation energy for ketonisation. The TOF of ∼13 min^−1^ is in excellent agreement with that of Red Mud (11.9 min^−1^) and sits in the middle of the values reported for the continuous vapour‐phase propanoic acid ketonisation over silica and heteropolyacid‐supported Pd, Pt and Cu nanoparticles (1.3–34 min^−1^) reported by Alotaibi et al.^50^ (although these values appear to have been determined under H_2_ and hence likely reflect HDO performance) and lower than those for the cross‐ketonisation of acetic and hexanoic acid over zeolites (50–100 min^−1^).^51^ There are a few studies on particle size effects in carboxylic acid ketonisation over oxide catalysts. For acetic acid ketonisation over nanocrystalline ceria, larger particles formed by high‐temperature calcination deliver higher activities but lower acetone yields, however, ceria crystallinity and not morphology was identified as the key factor.^46^ Propanoic acid ketonisation over nanocrystalline ceria is reportedly favoured over CeO_2_(1 1 1) facets and is dominant on larger particles and hence also structure‐sensitive, although propanal and 1‐propanol ketonisation were structure‐insensitive over the same materials. The origin of this different reactivity between Fe_3_O_4_ and ceria active phases requires further investigation.

The acetone selectivity determined under differential conditions was also size‐invariant at ∼60 % for all Fe_3_O_4_/SiO_2_ catalysts (Figure S11), which implies a common active (Lewis acid) site and is comparable to that reported over diverse metal oxides such as those of Ce, Fe, Mn, Ti, V and Zr.^8^, ^9^, ^48^, ^52^ Common by‐products such as CO, isobutene and acetaldehyde were not observed in this work, and only trace (<1 %) CH_4_ (as a primary product of acetic acid decarboxylation)^8^, ^53^ was detected alongside acetone, CO_2_ and water. Some coking was also observed, and the used catalysts contained ∼5 wt % carbon (determined by using elemental CHNS analysis). All catalysts were stable at each reaction temperature for 1 h, and indeed exhibited a minimal change in either conversion or selectivity upon holding for 8 h at the final 450 °C reaction temperature, however, extended ageing and recycling tests are the subject of future studies.

## Conclusions

Wet impregnation offers a simple means to prepare magnetite (Fe_3_O_4_) nanoparticles of varying size dispersed over fumed SiO_2_. The physico‐chemical properties of such silica‐supported Fe_3_O_4_ nanoparticles are largely size‐invariant and are characterised by weak/moderate‐strength Lewis acid sites that bind acetic acid in a bidentate, chelating acetate mode. Small Fe_3_O_4_ nanoparticles (∼6 nm diameter) afford high acid site densities as a result of their high dispersion and exhibit excellent conversions and mass‐normalised specific activities for the vapour‐phase acetic acid ketonisation to acetone at a reaction temperature between 300–450 °C. Ketonisation is structure‐insensitive over silica‐supported Fe_3_O_4_, which exhibits a catalytic performance comparable to that of industrial Red Mud; nanoparticulate Fe_3_O_4_ appears to be the principal active component of Red Mud waste for acetic acid ketonisation. This study paves the way to a deeper understanding of the catalytic properties and wider application of this abundant waste material.

## Experimental Section

**Materials**: Silica‐supported magnetite particles of varying sizes were prepared by the wet impregnation of fumed silica with iron nitrate. Briefly, a suspension of fumed SiO_2_ (Sigma–Aldrich, S5505) was stirred in EtOH at 40 °C for 30 min before the addition of an appropriate volume of an ethanolic solution of Fe(NO_3_)_3_⋅9 H_2_O to achieve iron loadings of 4–63 wt %. The slurry was stirred and evaporated to dryness at 50 °C, and the resulting solid was dried at 80 °C, ground to a fine powder (60 mesh) and calcined in air in a muffle furnace at 400 °C for 2 h. The resulting orange powder was subsequently reduced in a tube furnace under flowing H_2_ (10 mL min^−1^) at 350 °C for 30 min to obtain the desired Fe_3_O_4_ phase (as a grey/black powder).

**Catalyst characterisation**: Nitrogen physisorption was performed by using a Quantachrome Nova 1200 porosimeter, and samples were degassed at 120 °C in vacuo for 4 h before the adsorption/desorption isotherms were recorded. The surface areas were determined by multipoint BET analysis. Power XRD patterns were collected by using a Bruker D8 Advance fitted with a LynxEYE high‐speed strip detector and CuK_α_ (1.54 Å) radiation, and a 0.2 mm Ni filter to remove K_β_ radiation. Crystallite sizes were estimated by peak width analysis using the Scherrer equation. XPS was undertaken by using a Kratos Axis HSi spectrometer fitted with a charge neutraliser and magnetic focusing lens that employed AlK_α_ monochromatic radiation (1486.7 eV). Spectral fitting was performed using CasaXPS version 2.3.14. Binding energies were corrected to the C 1s peak at 284.6 eV, and surface atomic compositions were calculated by correction for the appropriate instrument response factors. In situ XRD patterns were obtained by using an Anton Parr XK900 cell interfaced to back‐pressure regulated Bronkhorst ELFLOW mass flow controllers. DRIFTS was performed by using a Nicolet iS50 FTIR spectrometer by using a Harrick Scientific Praying Mantis High‐Temperature Reaction Chamber and associated temperature controller. The acid character was evaluated from pyridine chemisorption. Iron oxide samples were wetted with pyridine (∼0.2 mL) and dried in a vacuum oven at 40 °C overnight before dilution to 10 wt % in dry KBr and spectra recorded in vacuo at 50 °C. Acetic acid adsorption was probed after RT pre‐saturation (∼0.2 mL) and subsequent evaporation to dryness in vacuo at 40 °C overnight. Samples were diluted to 10 wt % in dry KBr, and their spectra were recorded in vacuo between 50 and 400 °C; spectra of the untreated iron oxide samples diluted to 10 wt % in dry KBr were used to perform a background subtraction to obtain the adsorbate bands. TPRS of propylamine‐saturated samples was employed to calculate the acid site densities by using a Mettler Toledo TGA/DSC2 STARe system. Catalyst samples were pre‐saturated with propylamine (∼0.2 mL) at RT and evaporated to dryness in vacuo at 40 °C overnight. Samples (∼15 mg) were then heated in the TGA furnace to 800 °C at 10 °C min^−1^ under flowing N_2_ (40 mL min^−1^), and the evolved gases were analysed by using a Pfeiffer Vacuum Thermostar mass spectrometer to monitor the appearance of reactively formed propene over the acid sites. The resulting temperature‐programmed desorption spectra were background corrected for contributions from physisorbed propylamine on the silica support. TEM was performed by using a JEOL 2010 microscope operated at 200 kV. Images were collected by using a Gatan Ultrascan 4000 digital camera. Samples were dispersed in ethanol and deposited on 300 mesh carbon‐supported copper grids and dried in air. Particle diameters were measured with ImageJ software, and size distributions are based on the analysis of ∼150 particles for each sample. The bulk Fe content was determined by using inductively coupled plasma optical emission spectroscopy (ICP‐OES) by using a Thermo iCAP 7000 ICP‐OES instrument. Fe K‐edge transmission XAS was performed at the XAFS beamline of the Elettra synchrotron by using a Si(1 1 1) double‐crystal monochromator and ring operation at 250 mA/2 GeV.

**Ketonisation**: The catalytic ketonisation of acetic acid was performed in a continuous‐flow, packed‐bed microreactor with online GC analysis. The reactor comprised a 1 cm o.d. quartz tube, within which the catalyst bed was placed centrally and retained by quartz wool plugs. A constant catalyst bed volume of 4 cm^3^ was used in all experiments, which comprised approximately 50 mg each of Fe_3_O_4_ and SiO_2_ diluted with fused silica granules. The reactor tube was positioned in a temperature‐programmable furnace with a thermocouple placed in contact with the catalyst bed. Acetic acid was fed in a down‐flow fashion into the reactor by using an Agilent 1260 Infinity Isocratic Pump and N_2_ as the carrier gas (50 mL min^−1^) supplied by using a Brooks mass flow controller. All reactor lines were heated to 130 °C to prevent condensation, and a 1 cm diameter metal tube packed with fused silica granules was used to ensure acetic acid vaporisation before the reactor. For product stream analysis, we employed a Varian 3800 GC with a heated gas‐sampling valve and a BR‐Q PLOT column (30 m×0.53 mm i.d.) with a N_2_ carrier. Acetone, acetic acid and methane were detected by using a flame ionisation detector (FID) and CO_2_ was detected by using a thermal conductivity detector (TCD).

## Supporting information

As a service to our authors and readers, this journal provides supporting information supplied by the authors. Such materials are peer reviewed and may be re‐organized for online delivery, but are not copy‐edited or typeset. Technical support issues arising from supporting information (other than missing files) should be addressed to the authors.

Supplementary
