# Supplementary material for: Acetic Acid Ketonization over Fe3O4/SiO2 for Pyrolysis Bio‐Oil Upgrading
Source: ChemCatChem. 2017 Jan 18;9(9):1648–54. doi: 10.1002/cctc.201601269 (PMC5434921; doi:10.1002/cctc.201601269)
Supplement: Supplementary file 1 — Supplementary [file CCTC-9-1648-s001.pdf]

Heterogeneous & Homogeneous & Bio- & Nano-
CHEMCATCHEM
CATALYSIS

Supporting Information

Acetic Acid Ketonization over Fe_3O_4/SiO_2 for Pyrolysis Bio-Oil Upgrading

James A. Bennett,[a] Christopher M. A. Parlett,[a] Mark A. Isaacs,[a] Lee J. Durndell,[a] Luca Olivi,[b] Adam F. Lee,*[a] and Karen Wilson*[a]

cctc_201601269_sm_miscellaneous_information.pdf

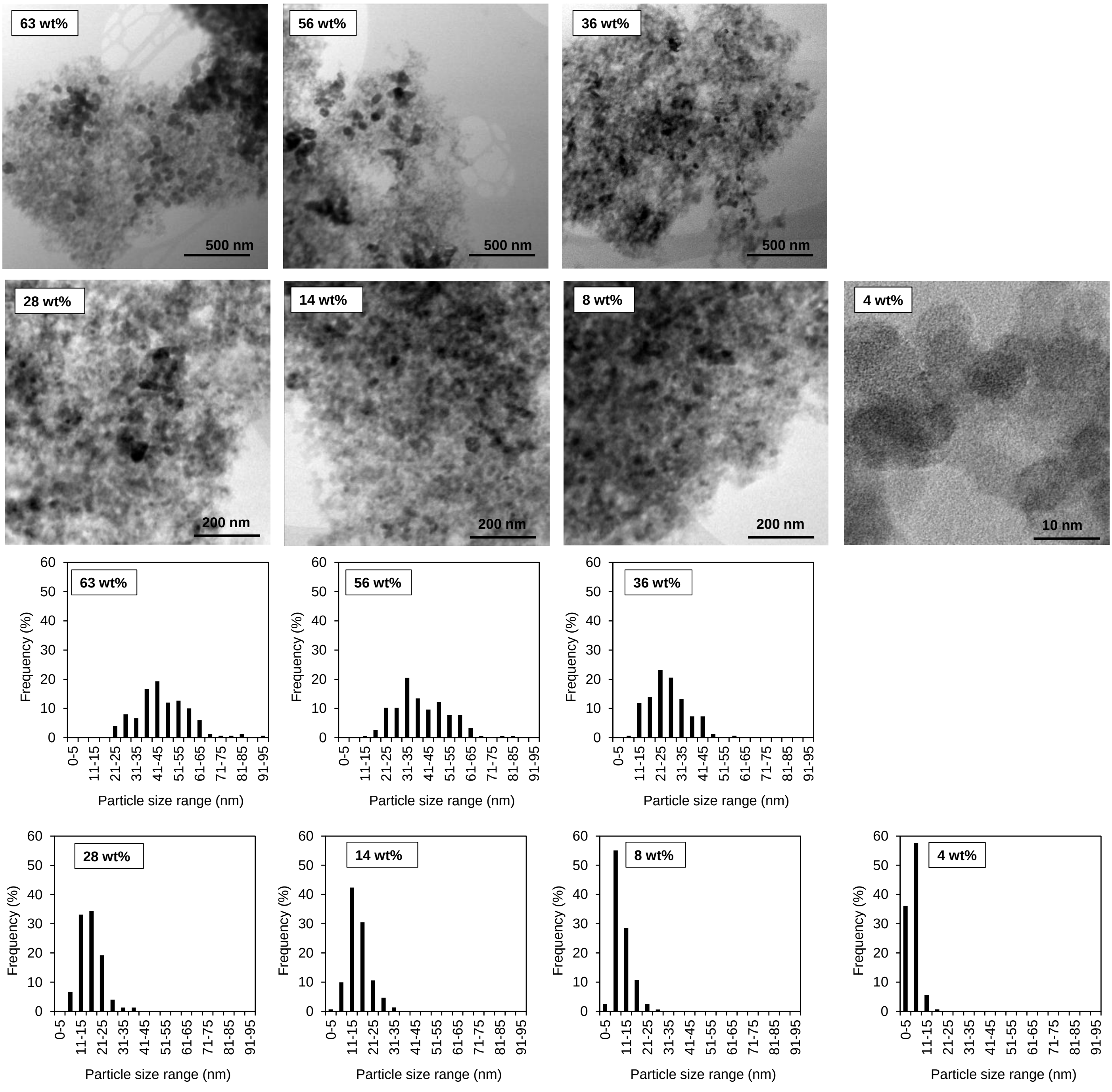

Figure S1. (top) Bright-field TEM images, and (bottom) particle size distributions for Fe_3O_4/SiO_2 catalysts.

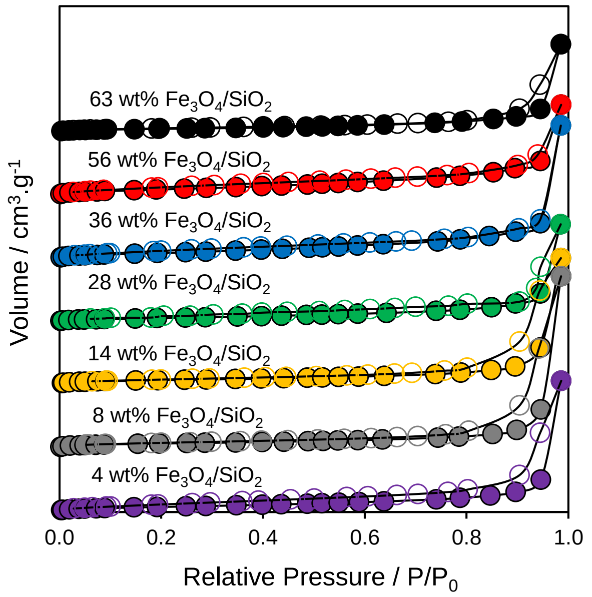

Figure S2. N_2 adsorption-desorption isotherms for Fe_3O_4/SiO_2 catalysts. Adsorption isotherms are represented by solid markers and desorption isotherms by hollow markers.

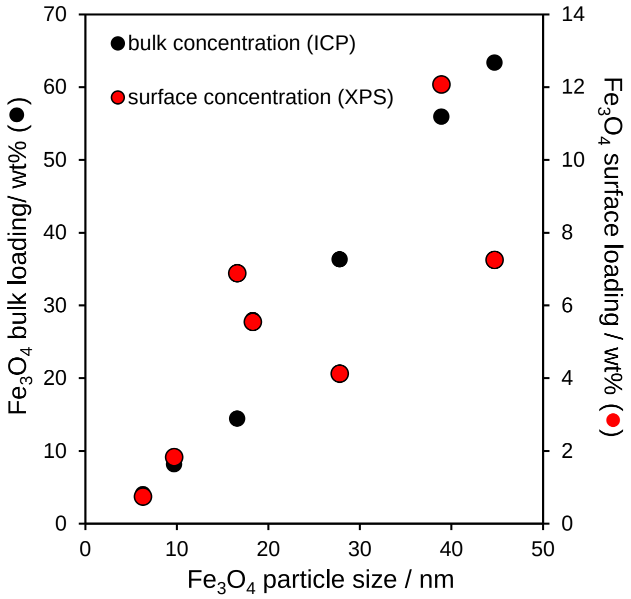

Figure S3. Surface and bulk Fe_3O_4 loadings as a function of particle size (determined by XRD) for Fe_3O_4/SiO_2 catalysts.

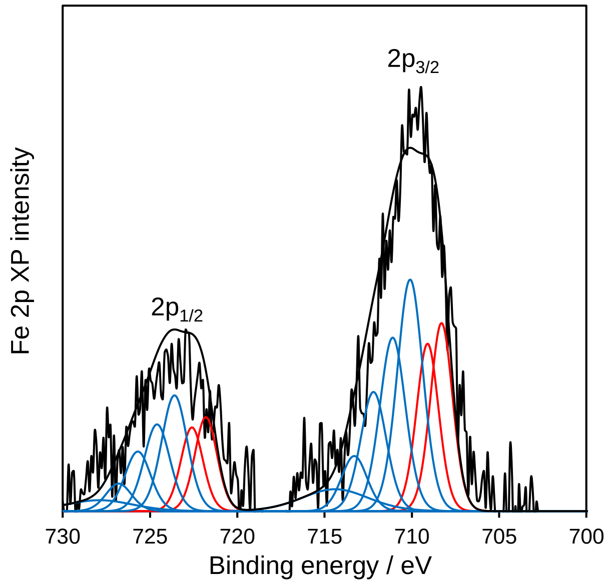

Figure S4. Fitted Fe 2p XP spectra for 63 wt% Fe_3O_4/SiO_2. Experimental data is shown in grey, fitted curve in black and Fe^{2+} and Fe^{3+} components in red and blue respectively.

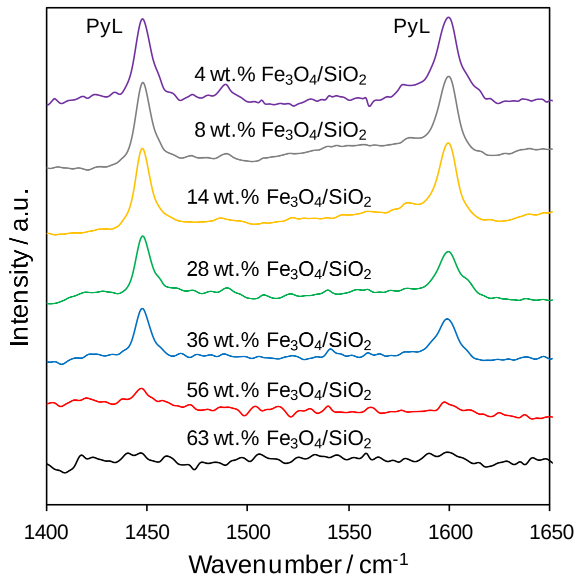

Figure. S5. DRIFT spectra of chemisorbed pyridine over Fe_3O_4/SiO_2 catalysts recorded at 50 °C in vacuo. Bands labelled PyL arise from pyridine adsorbed over Lewis acid sites.

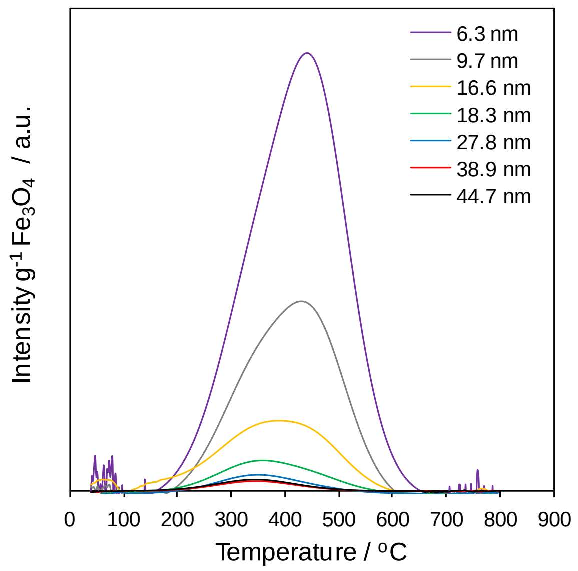

Figure S6. TPD of reactively-formed propene (m/z 41 channel) from chemisorbed propylamine normalised to the mass of Fe_3O_4, and background subtracted to remove contributions from molecular propylamine adsorbed on the silica support.

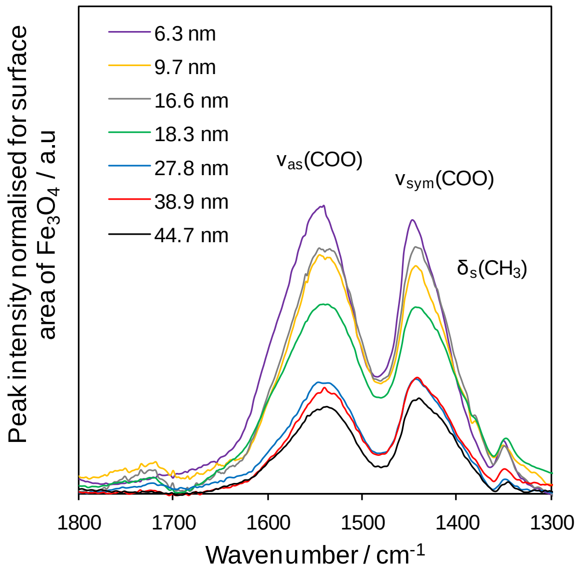

Figure S7. DRIFT spectra of room temperature adsorbed acetic acid over Fe_3O_4/SiO_2 catalysts normalised to the Fe_3O_4 surface area (geometric surface areas were estimated from the Fe_3O_4 particle diameter determined by HRTEM, assuming a spherical morphology). Spectra recorded at 200 °C in vacuo to remove physisorbed acid contributions.

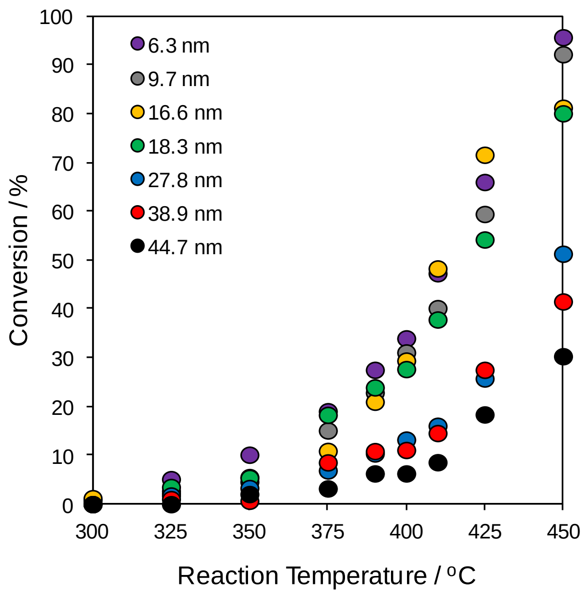

Figure S8. Acetic acid conversion over Fe_3O_4/SiO_2 catalysts of varying Fe_3O_4 particle size at increasing reaction temperatures. Values reported are the average after 1 h reaction at each temperature.

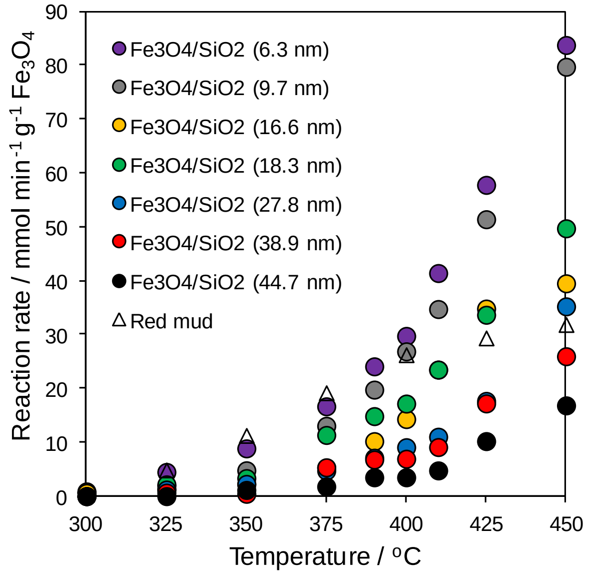

Figure S9. Rate of acetic acid conversion over Fe_3O_4/SiO_2 and Red Mud. Data is normalised for the mass of Fe_3O_4 present in the catalyst. Values reported are the average after 1 h reaction at each temperature.

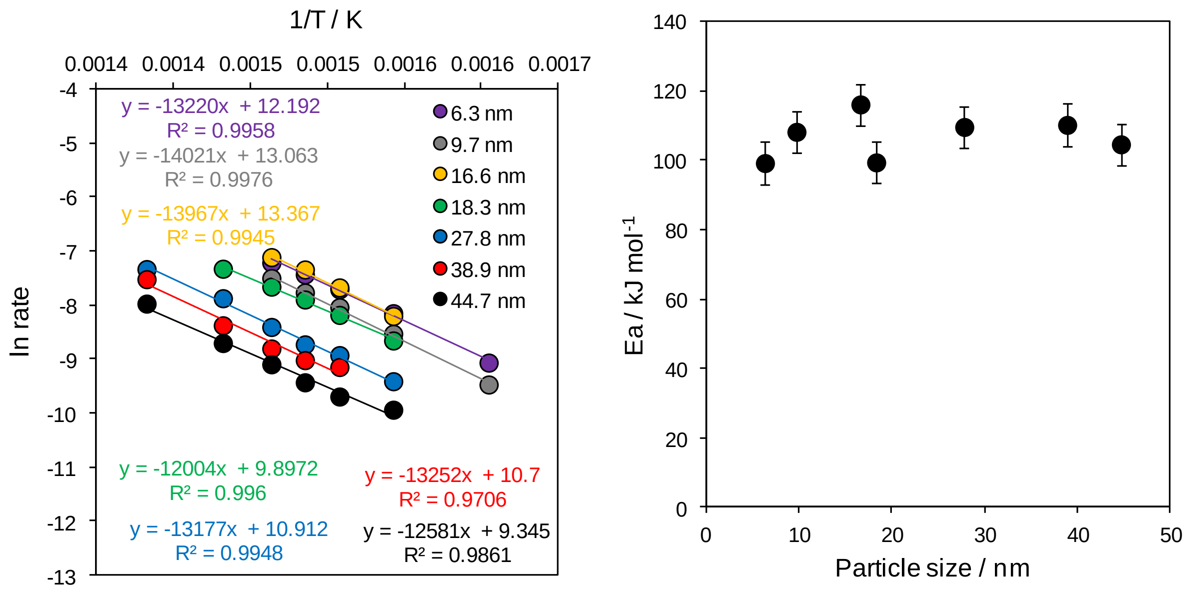

Figure S10. Arrhenius plots (left) and activation energies (right) for acetic acid ketonisation over Fe_3O_4 of increasing particle size. Values are calculated using data corresponding to conversions of 10-50 %.

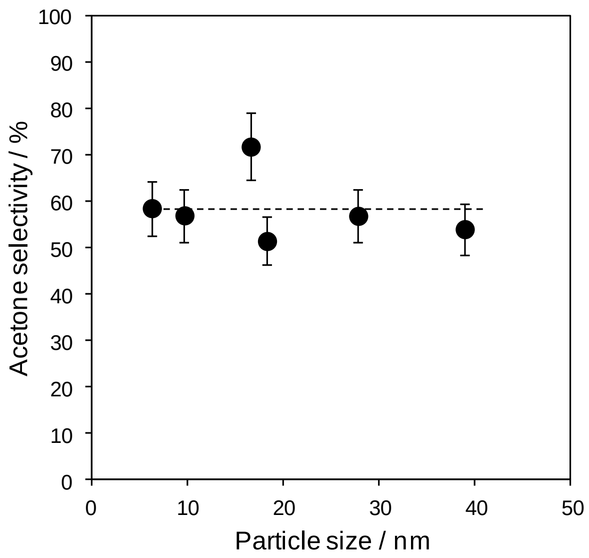

Figure S11. Acetone selectivity vs Fe_3O_4 particle size at an acetic acid conversion of ~50 %.

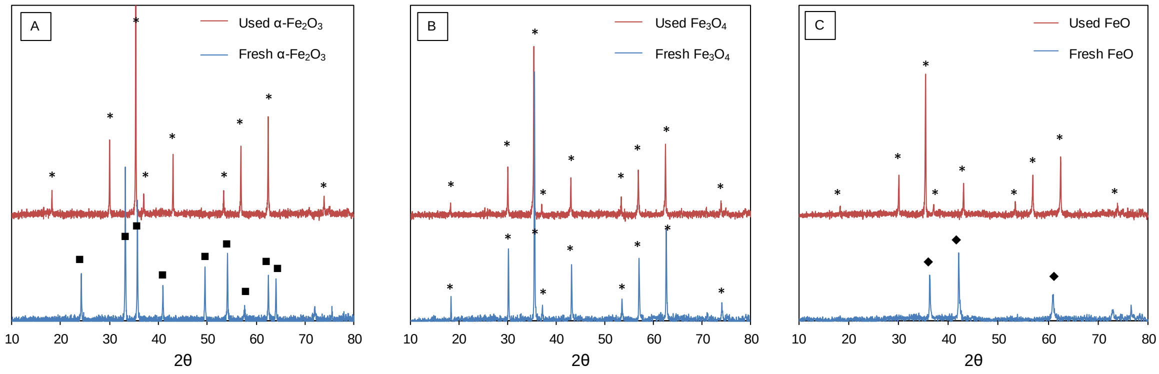

Figure S12. XRD patterns of fresh and used Fe_2O_3, Fe_3O_4 and FeO ketonisation catalysts. Diffraction peaks corresponding to α-Fe_2O_3, Fe_3O_4 and FeO phases are indicated with squares (■) asterisks (*) and diamonds (◆) respectively. All used catalysts showed only peaks corresponding to Fe_3O_4.

Table S1. Estimated Fe_3O_4 surface area for Fe_3O_4/SiO_2 catalysts.[a]

Fe_3O_4 loading / wt%	4	8	14	**28**	36	56	63
Fe_3O_4 surface area / $m^2.g_{catalyst}^{-1}$	7.4	9.7	10.1	**17.7**	15.1	16.7	16.4
Acid site density / $mmols.g_{catalyst}^{-1}$	0.169	0.199	0.256	**0.288**	0.22	0.251	0.252

[a]Assuming spherical particles with diameters from XRD as reported in Table 1 of the main text.

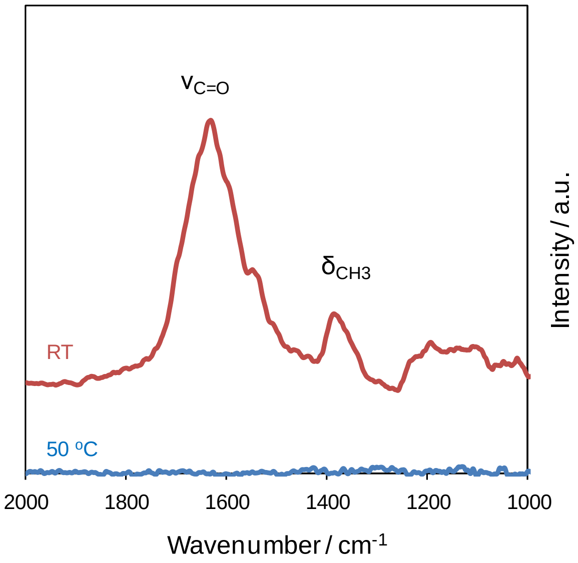

Figure S13. DRIFT spectra of chemisorbed acetone over pure magnetite at room temperature and 50 °C. Bands for molecular acetone are visible at 1631 and 1387 cm^{-1}.
